# *De novo* transcriptome assembly and positive selection analysis of an individual deep-sea fish

**DOI:** 10.1186/s12864-018-4720-z

**Published:** 2018-05-24

**Authors:** Yi Lan, Jin Sun, Ting Xu, Chong Chen, Renmao Tian, Jian-Wen Qiu, Pei-Yuan Qian

**Affiliations:** 1 Department of Ocean Science and Division of Life Science, Hong Kong University of Science and Technology, Clear Water Bay, Kowloon, Hong Kong, China; 2 Department of Biology, Hong Kong Baptist University, Hong Kong, China; 3 Japan Agency for Marine-Earth Science and Technology (JAMSTEC), 2-15 Natsushima-cho, Yokosuka, Kanagawa 237-0061 Japan

**Keywords:** Positive selection, High hydrostatic pressure, Cold shock, Cytoskeleton, Microtubule

## Abstract

**Background:**

High hydrostatic pressure and low temperatures make the deep sea a harsh environment for life forms. Actin organization and microtubules assembly, which are essential for intracellular transport and cell motility, can be disrupted by high hydrostatic pressure. High hydrostatic pressure can also damage DNA. Nucleic acids exposed to low temperatures can form secondary structures that hinder genetic information processing. To study how deep-sea creatures adapt to such a hostile environment, one of the most straightforward ways is to sequence and compare their genes with those of their shallow-water relatives.

**Results:**

We captured an individual of the fish species *Aldrovandia affinis*, which is a typical deep-sea inhabitant, from the Okinawa Trough at a depth of 1550 m using a remotely operated vehicle (ROV). We sequenced its transcriptome and analyzed its molecular adaptation. We obtained 27,633 protein coding sequences using an Illumina platform and compared them with those of several shallow-water fish species. Analysis of 4918 single-copy orthologs identified 138 positively selected genes in *A. affinis*, including genes involved in microtubule regulation. Particularly, functional domains related to cold shock as well as DNA repair are exposed to positive selection pressure in both deep-sea fish and hadal amphipod.

**Conclusions:**

Overall, we have identified a set of positively selected genes related to cytoskeleton structures, DNA repair and genetic information processing, which shed light on molecular adaptation to the deep sea. These results suggest that amino acid substitutions of these positively selected genes may contribute crucially to the adaptation of deep-sea animals. Additionally, we provide a high-quality transcriptome of a deep-sea fish for future deep-sea studies.

**Electronic supplementary material:**

The online version of this article (10.1186/s12864-018-4720-z) contains supplementary material, which is available to authorized users.

## Background

The deep sea is characterized by high hydrostatic pressure, darkness and low temperatures [1]. Among these characteristics, high hydrostatic pressure is regarded as the harshest for living organisms, since it can inhibit the functions of proteins through denaturing and impairing their structures [2, 3]. This is especially for enzymes [4, 5] and cytoskeleton proteins [6]. Besides, at low temperatures, DNA and RNA strands tend to tighten their structures, hindering the involvement of enzymes in DNA replication, transcription and translation [7] and thus disrupting the transcription and translation processes.

In eukaryotes, actin and microtubules are the primary constituents of cytoskeleton organization, which contributes to maintaining cytoskeletal structures, intracellular transport and cell motility [8]. However, high hydrostatic pressure has been found to disrupt actin fibers, microtubules and myosins in mammalian cells [6]. High hydrostatic pressure can also influence the cellular regulatory system, which controls and regulates the cytoskeletal changes, thus disrupting the assembly of actin filaments and microtubules [9]. Therefore, high hydrostatic pressure can affect all sorts of biological processes that rely on the cytoskeleton, such as spindle formation, cell division, mitosis and meiosis [10].

As a response to high hydrostatic pressure, cells in deep-sea organisms develop counteractive strategies, such as amino acid substitutions at specific key sites of actin sequences [8, 11] to help stabilize the advanced structures of proteins. For instance, Q137K and A155S can maintain the coupling of ATP and Ca^2+^ to counteract the dissociation effects of high pressure, and both V54A and L67P can help sustain the DNase I activity [8, 11]. A few amino acid substitutions of lactate dehydrogenase from deep-sea fish were suggested to help the enzyme better tolerate and function under high hydrostatic pressure [12]. Amino acid substitutions may also contribute to the hydrostatic pressure adaptation of protein-protein interactions or ligand binding [13]. In fact, this is not limited to deep-sea fish. In the amphipod *Hirondellea gigas*, amino acid substitutions likely provide the main resource for molecular adaptation, allowing this creature to survive and thrive in hadal trenches [14]. Additionally, osmolytes can help proteins fold properly and remain stable so that they can maintain their functions under high hydrostatic pressure [15, 16].

*Aldrovandia affinis* (Günther, 1877) [17] is a benthopelagic teleost fish (Actinopterygii: Teleostei) commonly found in the deep sea. It has a snake-like body and a pointed snout. This species is widespread in the Atlantic Ocean and Pacific Ocean at depths ranging from 730 m to 2560 m [18]. To adapt to a wide range of hydrostatic pressure, *A. affinis* has likely developed capabilities to maintain protein structures and functions, especially cytoskeleton organization [2], but little is known about the molecular mechanisms.

Genome-wide patterns of positive selection can be effectively identified through transcriptome sequencing combined with a branch site model [19–21]. In this approach, proteins of a specific species are compared with its ancestral protein sequences predicted through phylogeny. If the nonsynonymous substitution rate (dN) is significantly larger than the synonymous substitutions rate (dS), the genes are defined as positively selected [22, 23]. In the present study, the transcriptome of *A. affinis* captured from the Okinawa Trough at a depth of 1550 m using a ROV was sequenced and compared with those of three shallow-water fish species (the cave fish *Astyanax mexicanus*, the cod fish *Gadus morhua*, and the platy fish *Xiphophorus maculatus*) in order to identify positively selected genes.

## Methods

### Sample collection, RNA extraction, and sequencing

**Fig. 1:**
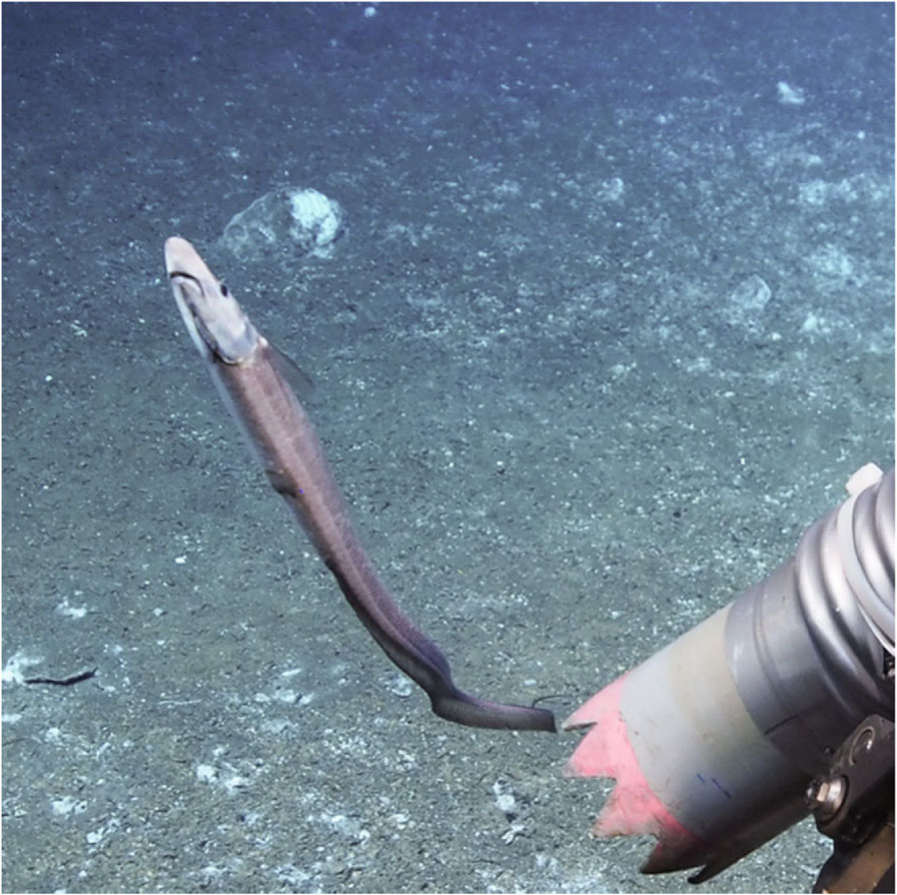
Photograph of an individual of the deep-sea fish *Aldrovandia affinis*. *Aldrovandia affinis* being captured from the Sakai hydrothermal vent field of the Okinawa Trough (21°31.4749’ N, 126°59.021′ E) at a depth of 1550 m by the remotely operated vehicle *Kaiko* (Dive Number #676)

During the Japan Agency for Marine-Earth Science and Technology (JAMSTEC) R/V *Kairei* cruise KR15–17 in November 2015, an individual *A. affinis* (JAMSTEC sample no. 1150047615) (Fig. 1) was captured from the Sakai hydrothermal vent field [24, 25] of the Okinawa Trough (21°31.4749’ N, 126°59.021′ E) at a depth of approximately 1550 m by the ROV *Kaiko Mk-IV*. A section of muscle tissue was preserved in RNA*later* at 4 °C overnight, and transferred to − 80 °C afterwards. The species was identified initially based on morphology [18] and later confirmed with the COI barcoding sequence. RNA was extracted using the TRIzol Reagent (Invitrogen, USA) according to the manufacturer’s instruction. The quality and quantity of RNA were evaluated by 1.5% agarose gel electrophoresis and with the NanoDrop 2000 (Thermo). The RNA quality was further tested with the Agilent 2100 Bioanalyzer and the RNA integrity number was 7.8. A full-length cDNA library was constructed and sequenced on the Illumina HiSeq 4000 with the read length of 150 bp.

### Data filtering, *de novo* assembly, functional annotation and reference species determination

Trimmomatic version 0.33 [26] was used to trim the adaptors and remove low-quality reads. Trinity version 2.0.6 [27] was utilized to *de novo* assemble all filtered reads. Due to redundancy of isoforms generated during alternative splicing, only the isoform with the highest abundance as estimated by RSEM was retained for each gene. CD-HIT-EST version 4.6.5 [28] with the setting of “-c 0.95” was used to remove transcripts whose sequence similarity exceeded 95% [14, 29]. BUSCO version 2.0 [30] and the metazoa_odb9 database were used to assess the completeness of the non-redundant transcripts. Coding sequences of the non-redundant transcripts were then predicted and translated using TransDecoder [27, 31] with a cut-off of 100 bp as recommended by the Trinity manual [27]. Each transcript was represented by the longest translated protein sequence in subsequent analyses.

All translated protein sequences were compared to sequences in NCBI non-redundant (NR) database using BLASTp version 2.2.31 with an *E*-value < 1 × e^− 5^. Then, the NR hits of all protein sequences were classified according to the taxonomy database of NCBI using MEGAN5 [32]. The functions and annotations of proteins were predicted with Blast2GO version 3.1 [33] to search against the Gene Ontology (GO) database. The KEGG (Kyoto Encyclopedia of Genes and Genomes) Automatic Annotation Server [34] was used together with the bi-directional BLAST method to identify pathway information. Subsequently, the GO item distribution for biological process, cellular components and molecular functions was summarized and plotted with WEGO version 1.0 [35].

### Amino acids and codons usage analysis

The codon usage of the overall coding region was calculated in CodonW version 1.4.4 [36]. The codon usage bias was determined by the relative synonymous codon usage (RSCU). RSCU > 1 and RSCU < 1 imply positive and negative codon usage bias, respectively. If RSCU equals 1, the codon usage is regarded as no bias. A Perl script was written to calculate the proportion of amino acids.

### Identification of orthologs and phylogenetic analysis

Single-copy orthologs shared by the deep-sea fish *A. affinis* and sequences of shallow-water fishes *Astyanax mexicanus*, *Gadus morhua*, *Lepisosteus oculatus*, *Oryzias latipes*, *Tetraodon nigroviridis*, *Xiphophorus maculatus* and *Latimeria chalumnae*, in the Ensembl database were identified using OrthoMCL version 2.0.9 [37] relying on all-vs-all BLASTp with an *E*-value threshold of 1 × e^− 5^ and MultiParanoid [38] which clusters pairwise orthologs inferred with InParanoid [39]. Aligned amino acid sequences of single-copy orthologs between *A. affinis* and the shallow-water species (with *Latimeria chalumnae* serving as the outgroup; class Sarcopterygii) were concatenated and used for constructing a phylogenetic tree using RAxML version 8.2.4 [40], which applies maximum-likelihood analysis based on the substitution model of PROGAMMA + GTR with 100 bootstraps. The phylogenetic tree with the highest bootstrap value was used in subsequent positive selection analysis. To exclude the influences of paralogs generated from genome duplication within the species, single-copy orthologs derived from speciation were used. Indeed, including a greater number of the shallow-water species would increase the statistical significance of the result. However, doing so would also reduce the number of common single-copy orthologs. Therefore, there is a trade-off between the number of shared single-copy orthologs and the number of species used. Moreover, genome duplication is common in fish [41, 42] and also reduces the number of single-copy orthologs. We ran several trials and found that three particular shallow-water fish species (*A. mexicanus*, *G. morhua*, and *X. maculatus*) can provide the subsequent positive selection analysis with the greatest number of single-copy orthologs. Therefore, these three species of fish were referred to the subsequent positive selection analysis of *A. affinis*.

### Positive selection analysis

The same analytical pipeline described in the previous genome study [29] was used to identify positively selected genes in the deep-sea *A. affinis*. A modified branch site model A [43] coupled with Bayesian Empirical Bayes (BEB) methods [44] was adopted to compare *A. affinis* with the shallow-water species.

MUSCLE [45] was used to align amino acid sequences, and amino acid alignment further guided the alignment of coding DNA sequences in ParaAT version 1.0 [46] with the “-g” flag to delete gaps in the aligned sequences. The strength of positive selection on each codon of each orthologous gene along a specific targeted lineage of a phylogenetic tree, designated as the deep-sea *A. affinis*, was estimated with the modified branch site model using *codeml* of the PAML package [47]. To determine to what degree these codon sequences along the targeted lineage fit the branch site model including positive selection better than the one containing neutral selection or negative selection, an alternative branch site model (Model = 2, NSsites = 2 and Fix = 0) and a neutral branch site model (Model = 2, NSsites = 2, Fix = 1 and Fix ω = 1) were combined to calculate log-likelihood values for each model using likelihood ratio tests. The log-likelihood values generated were used to assess the model fit, using the Chi-square test with one degree of freedom [43]. A multiple testing correction method [48] was then applied to correct the *P* values. In addition, potential positive selection of codon sites was assessed by their posterior probabilities calculated with the BEB method. If the posterior probability exceeds 0.9, then the amino acid site would be considered as a positively selected site. Genes with an adjusted *P* value < 0.1 [49, 50] and positively selected amino acid sites were regarded as positively selected genes.

## Results

**Fig. 2:**
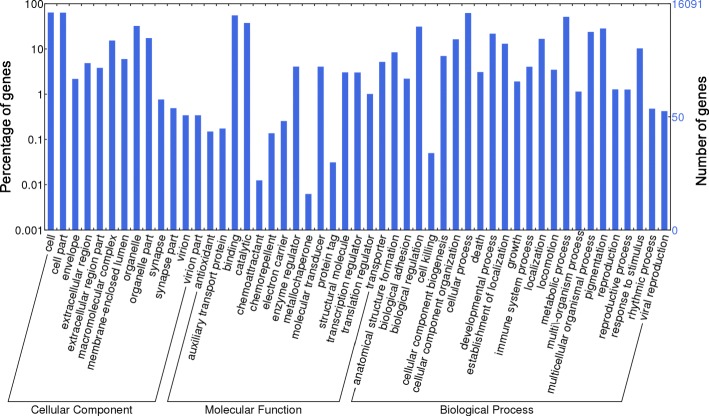
Gene ontology distribution for the cellular component, molecular function and biological process of *Aldrovandia affinis*

A total of 38,370,894 raw paired-end reads (150 bp) were cleaned and filtered, resulting in 31,858,276 reads (83%) that were retained and used for *de novo* assembly. After removing redundant isoforms from the raw transcriptome assembly and predicting the open reading frame, 27,633 non-redundant transcripts ranging from 297 bp to 19,469 bp had a total size of 27,427,719 bp and a contig N50 value of 1359 nt (Table 1). The length distribution of the assembled contigs is shown in Additional file 1: Figure S1. These non-redundant transcripts hit 90.9% of the single-copy orthologs in the BUSCO metazoan database, including 82.2% of the complete orthologs and 8.7% of the fragmented orthologs. Translating all of these *A. affinis* transcripts with the open reading frames, 23,196 (~ 84%) of these sequences were significantly matched to the existing protein sequences in the NCBI NR database; 15,999 had at least one significant match to the GO item; and 7954 had significant hits in terms of KEGG pathways (Table 1). The GO item distribution of *A. affinis* (Fig. 2) for biological processes, molecular functions and cellular components did not appear to be significantly biased, indicating that there was no sequence bias in the reads.

**Table 1:** Statistics of assembly and annotation for *Aldrovandia affinis*

Trinity assembly	*Aldrovandia affinis*
Sequencing data	5,755,634,100 bp
Total number of coding transcripts	27,633
Total assembled nucleotide bases	27,427,719 bp
N50 length	1359 nt
Annotation	
Total translated proteins	25,851
NR	23,196 (90%)
GO	15,999 (62%)
KEGG	7954 (31%)

**Fig. 3:**
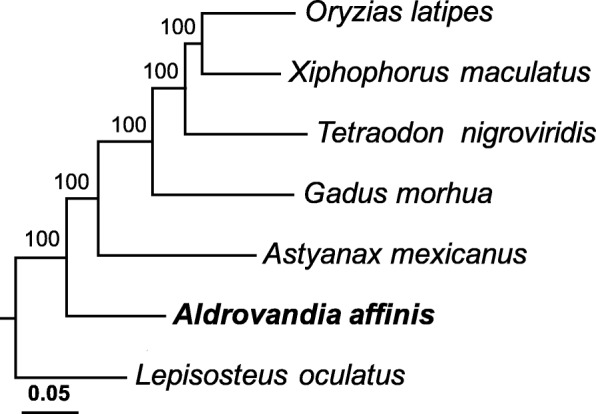
Maximum-likelihood phylogenetic tree for *Aldrovandia affinis* and shallow-water fish. The shallow-water fish species include the cave fish *Astyanax mexicanus*, the cod fish *Gadus morhua*, the spotted gar *Lepisosteus oculatus*, the medaka fish *Oryzias latipes*, the tetraodon fish *Tetraodon nigroviridis*, the platy fish *Xiphophorus maculatus* and the coelacanth *Latimeria chalumnae* (class Sarcopterygii; serving as the outgroup). This tree was constructed based on the substitution model of PROGAMMA + GTR with 100 bootstraps

**Fig. 4:**
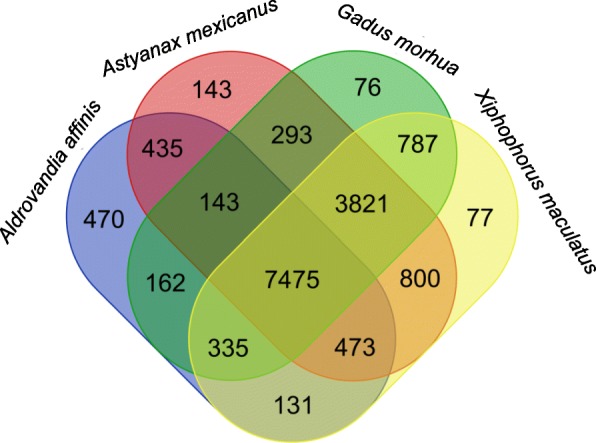
Gene families shared by *Aldrovandia affinis*, *Astyanax mexicanus*, *Gadus morhua* and *Xiphophorus maculatus*

A phylogenetic tree (Fig. 3) was constructed from a total of 349,341 amino acids that were aligned and trimmed from single-copy orthologs shared by the deep-sea fish *A. affinis* and shallow-water fishes *A. mexicanus*, *G. morhua*, *L. oculatus*, *O. latipes*, *T. nigroviridis*, *X. maculatus* and *L. chalumnae* (the latter of which served as the outgroup). *A. mexicanus*, *G. morhua* and *X. maculatus* were chosen for positive selection analysis because they shared 4918 single-copy orthologs with *A. affinis*, which is the greatest number possible from this pool. The Venn diagram shows that 7475 gene families were shared among *A. affinis*, *A. mexicanus*, *G. morhua* and *X. maculatus*, including both single-copy orthologs and multi-copy paralogs (Fig. 4). There was no significant amino acid and codon usage bias among these four species (Additional file 1: Table S1). Among these orthologous genes, 138 genes (Additional file 1: Table S2) in *A. affinis* fitted the alternative branch site model significantly better assuming positive selection and had positively selected amino acid sites with a posterior probability exceeding 0.9. A set of proteins involved in cytoskeleton organization, especially proteins stabilizing actin and microtubules, and nucleic-binding proteins involved in genetic information processing had a clear positive sign (Table 2).

**Table 2:** Positively selected genes related to the cytoskeleton system and genetic information processing in *Aldrovandia affinis*

Gene	Function	Description	Adjusted *P* value
Cytoskeleton system			
COL18A2	collagen alpha-2(I) chain	Basement membrane	1.82E-04
COL18A1	collagen alpha-1 (XVIII) chain	Basement membrane	3.48E-02
C21orf2	protein C21orf2	Cytoskeleton organization	1.04E-02
DST	dystonin	Link actin	4.24E-08
GCP3	gamma-tubulin complex component 3	Microtubule nucleation	8.42E-02
CDK5RAP2	CDK5 regulatory subunit-associated 2	Microtubule nucleation	7.35E-02
PIKFYVE	1-phosphatidylinositol 3-phosphate 5-kinase	Actin regulation	3.20E-10
FRMD6	FERM domain-containing protein 6	Actin and cytoskeleton regulation	3.55E-02
CKAP5	cytoskeleton-associated 5	Cytoskeleton regulation	8.19E-02
RMDN1	regulator of microtubule dynamics 1	Microtubule Regulation	4.32E-02
CLASP2	CLIP-associating protein 2	Actin and microtubule stabilization	1.71E-02
FRYL	furry homolog-like	Microtubule stabilization	4.32E-02
AGTPBP1	cytosolic carboxypeptidase 1	Tubulin	1.03E-05
Genetic information processing			
ERCC4	DNA repair endonuclease XPF	DNA repair	1.55E-02
RFC1	replication factor C subunit 1	DNA replication	3.09E-02
DNAJC4	DnaJ homolog subfamily C member 4	Protein folding	1.20E-03
TAF1	transcription initiation factor TFIID subunit 1	Transcription	5.80E-06
GTF3C1	general transcription factor 3C polypeptide 1	Transcription	1.29E-04
TCEA1	transcription elongation factor A 3	Transcription	2.58E-02
HINFP	histone H4 transcription factor	Transcription	1.22E-02
DHX36	ATP-dependent RNA helicase DHX36	Transcription; cold shock	7.51E-03
EIF4B	eukaryotic translation initiation factor 4B	Translation	1.82E-06

## Discussion

High hydrostatic pressure and low temperatures are considered as two major barriers to survival in the deep sea [2, 7]. How certain animals cope with such adverse conditions remains largely unknown. A set of positively selected genes related to cytoskeleton structures, DNA repair and genetic information processing were identified in this study. This finding implies certain genes contribute to molecular adaptation to the deep sea. By comparing our results with those from our previous studies concerning the giant amphipod *H. gigas* collected from the Challenger Deep at a depth of approximately 11,000 m [14], we found that functional domains involved in separating the strands of the DNA double helix chain or the self-annealed RNA chain, including DEAD (Asp-Glu-Ala-Asp motif) box helicase, helicase conserved C-terminal domain, UvrD/REP helicase N-terminal domain and RNA helicase, as well as eukaryotic initiation factor 4G, are positively selected in both *A. affinis* and *H. gigas*. These domains are capable of generating cold shock response and are further involved in unwinding unfavorable secondary structures, which helps maintain the genetic information processing in the deep-sea environment [51, 52]. Both the fish *A. affinis* and the amphipod *H. gigas* are unable to regulate their body temperature themselves, which means that essential genetic processes such as DNA replication, transcription and translation, are confronted with the threats of low temperatures in the deep ocean [7, 53]. Moreover, high hydrostatic pressure treatment can trigger cold shock response in bacteria [54]. Therefore, cold shock genes are subjected to positive selection pressure, which may help animals deal with not only low temperatures but also high hydrostatic pressure to maintain their key genetic processes in the deep sea.

**Fig. 5:**
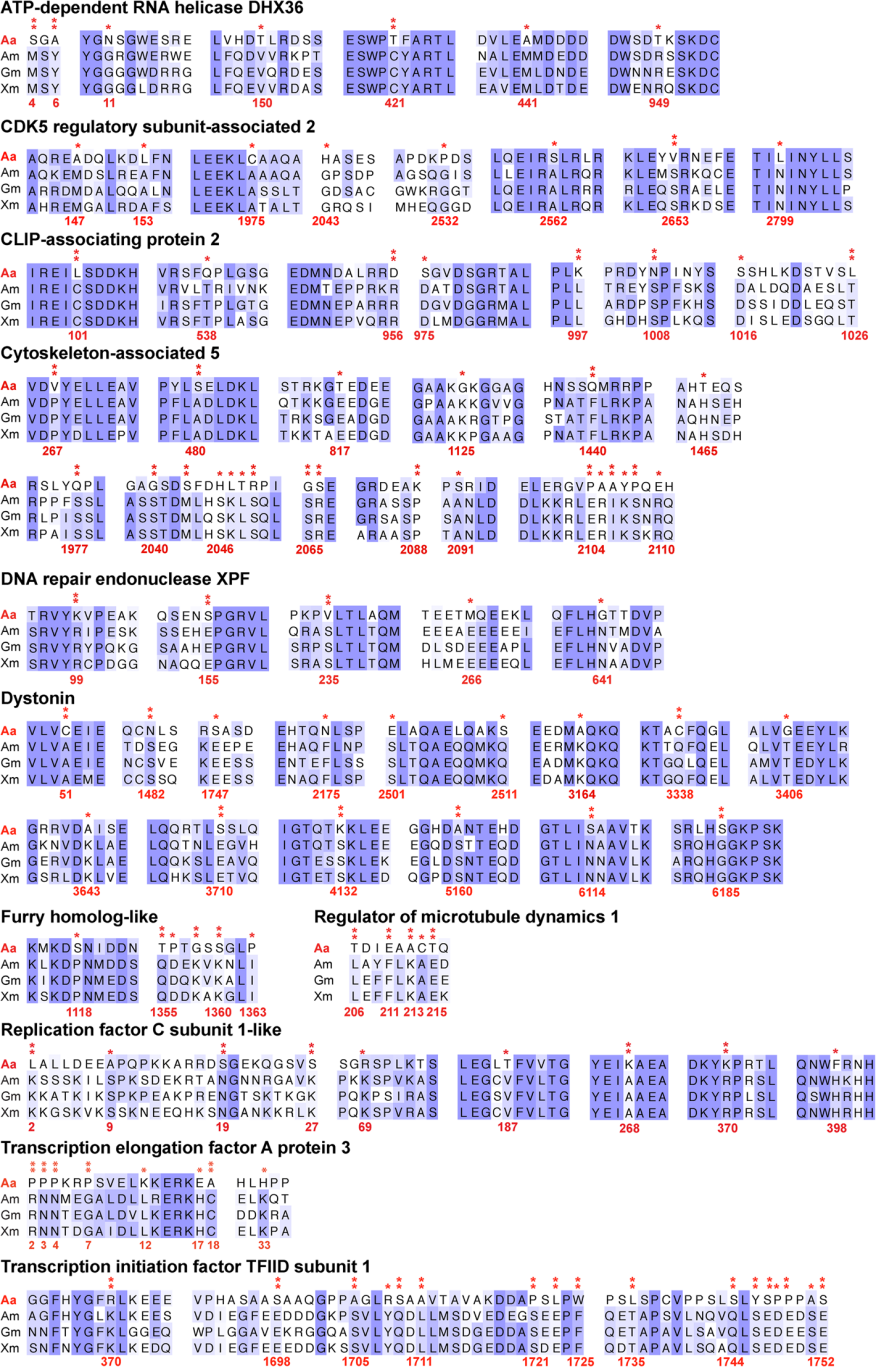
Partial alignment of positively selected genes. Double asterisks indicate that the amino acids in *Aldrovandia affinis* have a BEB posterior probability higher than 95% and a single asterisk indicates that the sites have a posterior probability between 90% and 95%. (Aa: *Aldrovandia affinis*; Am: *Astyanax mexicanus*; Gm: *Gadus morhua* and Xm: *Xiphophorus maculatus*)

Besides low temperatures, deep-sea organisms are exposed to high hydrostatic pressure that can cause DNA chain breakage and damage, and thus it is suspected that they would need to repair their DNA more frequently to maintain DNA integrity [55–58]. In the present study, two important genes involved in repairing DNA damage are positively selected in *A. affinis*. One is DNA repair endonuclease XPF (ERCC4) (Table 2, Fig. 5) that can contribute to repairing abnormal nucleotide excision and helping recombinant DNA remove cross-links during the homologous recombination stage [59]. The other gene is replication factor C subunit 1 (RFC1) (Table 2, Fig. 5). In the hadal amphipod *H. gigas*, replication factor A1 (RFA1) is positively selected [14]. Both RFC1 and RFA1 help repair DNA damaged by environmental stress [14, 60]. In the deep sea, animals cannot avoid exposure to high hydrostatic pressure, and their fundamental genetic information is vulnerable in such an extreme environment. Thus, deep-sea animals probably require stronger DNA repairing mechanisms to protect their genetic information from high hydrostatic pressure. The positive selection of genes required for DNA repair may be one of the reasons that deep-sea vertebrates and invertebrates can adapt to high hydrostatic pressure.

In contrast to the hadal amphipod *H. gigas* [14], a set of genes involved in cytoskeleton reorganization, especially microtubule regulation, are positively selected in the deep-sea fish *A. affinis*. The assembly of microtubules can be inhibited under high hydrostatic pressure [2, 9]. Microtubules determine the extension of axon formation and neuronal polarity [61], and thus one possible reason that these genes are associated with microtubule cytoskeletons under positive selection in the deep-sea fish *A. affinis* is that this particular fish has a much more developed nervous system than *H. gigas*. Genes involved in maintaining microtubules may be subjected to higher positive selection pressure to sustain the function of the nervous system under high hydrostatic pressure. Such positively selected genes include CDK5 regulatory subunit-associated 2 (CDK5RAP2), cytoskeleton-associated 5 (CKAP5) and CLIP-associating protein 2 (CLASP2) (Table 2, Fig. 5). These genes bind to the plus-end of microtubules to regulate the dynamics of their assembly [62–65]. Furthermore, CDK5RAP2 can promote microtubule nucleation in axons [66, 67]. Dystonin (DST) is a key protein linking F-actin and neuro-filaments to maintain neuronal cytoskeleton organization. The positive selection (Table 2, Fig. 5) of this protein may help protect the nervous system of deep-sea fish from the effects of high hydrostatic pressure [2, 68]. Even though the results obtained in the present study are based on one individual *A. affinis*, they still reflect the genetics of the entire species. This study has thoroughly compared the positive selection between deep-sea vertebrates and deep-sea invertebrates, which sheds light on the molecular adaptation of deep-sea animals.

## Conclusions

A set of positively selected genes related to cytoskeleton structures, DNA repair and genetic information processing were identified in the present study. These genes imply the molecular adaptation of animals to the deep sea. The deep-sea organisms rely on the amino acids substitutions of these positively selected genes as the main adaptation resources to survive in such an environment. Furthermore, the present study provides a high-quality, deep-sea transcriptome that can serve as a reference for future deep-sea studies.

## Additional file

Additional file 1: **Table S1.** Codon usage among *Aldrovandia affinis*, *Astyanax mexicanus*, *Gadus morhua* and *Xiphophorus maculatus*. **Table S2.** A complete list of positively selected genes in *Aldrovandia affinis*. **Figure S1.** Statistics of assembly contigs length. (DOCX 68 kb)

## Abbreviations

BEB: Bayesian Empirical Bayes
CDK5RAP2: CDK5 regulatory subunit-associated 2
CKAP5: Cytoskeleton-associated 5
CLASP2: CLIP-associating protein 2
DEAD: Asp-Glu-Ala-Asp motif
DST: Dystonin
ERCC4: DNA repair endonuclease XPF
GO: Gene ontology
JAMSTEC: Japan Agency for Marine-Earth Science and Technology
KEGG: Kyoto Encyclopedia of Genes and Genomes
LRT: Likelihood ratio tests
ML: Maximum-likelihood
NR: Non-redundant
RFA1: Replication factor A1
RFC1: Replication factor C subunit 1
ROV: Remotely operated vehicle
RSCU: Relative synonymous codon usage
