# Supplementary material for: De novo transcriptome assembly and positive selection analysis of an individual deep-sea fish
Source: BMC Genomics. 2018 May 24;19:394. doi: 10.1186/s12864-018-4720-z (PMC5968573; doi:10.1186/s12864-018-4720-z)
Supplement: Supplementary file 1 — Table S1. Codon usage among Aldrovandia affinis, Astyanax mexicanus, Gadus morhua and Xiphophorus maculatus. Table S2. A complete list of positively selected genes in Aldrovandia affinis. Figure S1. Statistics of assembly contigs length. (DOCX 68 kb) [file 12864_2018_4720_MOESM1_ESM.docx]

**Table S1.** Codon usage among *Aldrovandia affinis*, *Astyanax mexicanus*, *Gadus morhua* and *Xiphophorus maculatus*.

|  |  | ***A. affinis*** | | | ***A. mexicanus*** | | | ***G. morhua*** | | | ***X. maculatus*** | | |
| --- | --- | --- | --- | --- | --- | --- | --- | --- | --- | --- | --- | --- | --- |
|  |  | **Num** | **‰** | **RSCU** | **Num** | **‰** | **RSCU** | **Num** | **‰** | **RSCU** | **Num** | **‰** | **RSCU** |
| Phe | UUU | 70413 | 10.1 | 0.6 | 194815 | 15.9 | 0.9 | 81894 | 8.9 | 0.5 | 168017 | 15.1 | 0.8 |
|  | UUC | 164115 | 23.6 | 1.4 | 253550 | 20.8 | 1.1 | 260059 | 28.4 | 1.5 | 249334 | 22.3 | 1.2 |
| Leu | UUA | 20280 | 2.9 | 0.2 | 78841 | 6.5 | 0.4 | 22418 | 2.4 | 0.2 | 55100 | 4.9 | 0.3 |
|  | UUG | 60702 | 8.7 | 0.6 | 125059 | 10.2 | 0.6 | 70113 | 7.6 | 0.5 | 124686 | 11.2 | 0.7 |
|  | CUU | 49063 | 7.1 | 0.5 | 136405 | 11.2 | 0.7 | 57371 | 6.3 | 0.4 | 119499 | 10.7 | 0.7 |
|  | CUC | 143199 | 20.6 | 1.3 | 220188 | 18.0 | 1.1 | 218300 | 23.8 | 1.4 | 215012 | 19.3 | 1.2 |
|  | CUA | 31334 | 4.5 | 0.3 | 82516 | 6.8 | 0.4 | 41304 | 4.5 | 0.3 | 59211 | 5.3 | 0.3 |
|  | CUG | 353061 | 50.9 | 3.2 | 528636 | 43.3 | 2.7 | 510272 | 55.6 | 3.3 | 506077 | 45.4 | 2.8 |
| Ile | AUU | 64547 | 9.3 | 0.7 | 173209 | 14.2 | 1.0 | 58713 | 6.4 | 0.5 | 136482 | 12.2 | 0.8 |
|  | AUC | 177291 | 25.5 | 1.9 | 274118 | 22.4 | 1.5 | 281154 | 30.7 | 2.2 | 281543 | 25.2 | 1.7 |
|  | AUA | 35031 | 5.0 | 0.4 | 95364 | 7.8 | 0.5 | 44435 | 4.8 | 0.4 | 74312 | 6.7 | 0.5 |
| Met | AUG | 158316 | 22.8 | 1.0 | 281576 | 23.1 | 1.0 | 222220 | 24.2 | 1.0 | 261763 | 23.5 | 1.0 |
| Val | GUU | 53094 | 7.6 | 0.5 | 149568 | 12.2 | 0.8 | 59087 | 6.4 | 0.4 | 143430 | 12.9 | 0.8 |
|  | GUC | 118622 | 17.1 | 1.1 | 169767 | 13.9 | 0.9 | 180734 | 19.7 | 1.2 | 199369 | 17.9 | 1.1 |
|  | GUA | 30714 | 4.4 | 0.3 | 91699 | 7.5 | 0.5 | 33105 | 3.6 | 0.2 | 59729 | 5.4 | 0.3 |
|  | GUG | 234230 | 33.7 | 2.2 | 363716 | 29.8 | 1.9 | 333956 | 36.4 | 2.2 | 305356 | 27.4 | 1.7 |
| Ser | UCU | 76450 | 11.0 | 0.8 | 210312 | 17.2 | 1.2 | 78701 | 8.6 | 0.7 | 166957 | 15.0 | 1.0 |
|  | UCC | 160151 | 23.1 | 1.7 | 216827 | 17.8 | 1.2 | 222270 | 24.2 | 1.8 | 220869 | 19.8 | 1.4 |
|  | UCA | 56476 | 8.1 | 0.6 | 159925 | 13.1 | 0.9 | 55463 | 6.0 | 0.5 | 128271 | 11.5 | 0.8 |
|  | UCG | 57104 | 8.2 | 0.6 | 68579 | 5.6 | 0.4 | 88558 | 9.7 | 0.7 | 81765 | 7.3 | 0.5 |
|  | AGU | 59480 | 8.6 | 0.6 | 169681 | 13.9 | 1.0 | 62888 | 6.9 | 0.5 | 117553 | 10.5 | 0.7 |
|  | AGC | 164750 | 23.7 | 1.7 | 249837 | 20.5 | 1.4 | 219979 | 24.0 | 1.8 | 250607 | 22.5 | 1.6 |
| Pro | CCU | 83104 | 12.0 | 0.8 | 208653 | 17.1 | 1.2 | 81754 | 8.9 | 0.6 | 173086 | 15.5 | 1.1 |
|  | CCC | 184894 | 26.6 | 1.7 | 190029 | 15.6 | 1.1 | 238625 | 26.0 | 1.9 | 174228 | 15.6 | 1.1 |
|  | CCA | 83855 | 12.1 | 0.8 | 192651 | 15.8 | 1.1 | 80309 | 8.8 | 0.6 | 158398 | 14.2 | 1.0 |
|  | CCG | 79256 | 11.4 | 0.7 | 98763 | 8.1 | 0.6 | 112433 | 12.3 | 0.9 | 120446 | 10.8 | 0.8 |
| Thr | ACU | 59360 | 8.6 | 0.6 | 175922 | 14.4 | 1.0 | 55398 | 6.0 | 0.5 | 132463 | 11.9 | 0.9 |
|  | ACC | 155478 | 22.4 | 1.7 | 221071 | 18.1 | 1.3 | 238716 | 26.0 | 2.0 | 215866 | 19.3 | 1.4 |
|  | ACA | 76627 | 11.0 | 0.8 | 195085 | 16.0 | 1.1 | 75213 | 8.2 | 0.6 | 155195 | 13.9 | 1.0 |
|  | ACG | 77744 | 11.2 | 0.8 | 93301 | 7.6 | 0.5 | 120951 | 13.2 | 1.0 | 108050 | 9.7 | 0.7 |
| Ala | GCU | 90382 | 13.0 | 0.7 | 249410 | 20.4 | 1.3 | 95781 | 10.4 | 0.6 | 205955 | 18.5 | 1.1 |
|  | GCC | 225170 | 32.4 | 1.8 | 254141 | 20.8 | 1.3 | 331376 | 36.1 | 2.1 | 269198 | 24.1 | 1.5 |
|  | GCA | 85507 | 12.3 | 0.7 | 187896 | 15.4 | 1.0 | 80745 | 8.8 | 0.5 | 153126 | 13.7 | 0.8 |
|  | GCG | 91628 | 13.2 | 0.7 | 96602 | 7.9 | 0.5 | 131706 | 14.4 | 0.8 | 105847 | 9.5 | 0.6 |
| Tyr | UAU | 41025 | 5.9 | 0.5 | 125853 | 10.3 | 0.7 | 46219 | 5.0 | 0.4 | 93041 | 8.3 | 0.6 |
|  | UAC | 135034 | 19.5 | 1.5 | 216542 | 17.7 | 1.3 | 216013 | 23.5 | 1.7 | 213639 | 19.1 | 1.4 |
| His | CAU | 43165 | 6.2 | 0.5 | 116679 | 9.6 | 0.7 | 48944 | 5.3 | 0.4 | 97438 | 8.7 | 0.7 |
|  | CAC | 141167 | 20.3 | 1.5 | 207296 | 17.0 | 1.3 | 192755 | 21.0 | 1.6 | 190197 | 17.0 | 1.3 |
| Gln | CAA | 53975 | 7.8 | 0.3 | 124051 | 10.2 | 0.4 | 60603 | 6.6 | 0.3 | 116583 | 10.4 | 0.4 |
|  | CAG | 284175 | 40.9 | 1.7 | 454825 | 37.2 | 1.6 | 356679 | 38.9 | 1.7 | 407636 | 36.5 | 1.6 |
| Asn | AAU | 61159 | 8.8 | 0.5 | 173004 | 14.2 | 0.7 | 56368 | 6.1 | 0.3 | 128244 | 11.5 | 0.6 |
|  | AAC | 177502 | 25.6 | 1.5 | 298802 | 24.5 | 1.3 | 273045 | 29.8 | 1.7 | 304214 | 27.3 | 1.4 |
| Lys | AAA | 120751 | 17.4 | 0.6 | 313529 | 25.7 | 0.9 | 120851 | 13.2 | 0.5 | 270512 | 24.2 | 0.9 |
|  | AAG | 269050 | 38.8 | 1.4 | 389258 | 31.9 | 1.1 | 364653 | 39.8 | 1.5 | 365813 | 32.8 | 1.2 |
| Asp | GAU | 103369 | 14.9 | 0.6 | 264257 | 21.6 | 0.9 | 96516 | 10.5 | 0.4 | 205162 | 18.4 | 0.7 |
|  | GAC | 257616 | 37.1 | 1.4 | 347548 | 28.5 | 1.1 | 380718 | 41.5 | 1.6 | 372260 | 33.4 | 1.3 |
| Glu | GAA | 113936 | 16.4 | 0.5 | 271061 | 22.2 | 0.6 | 109581 | 11.9 | 0.4 | 247012 | 22.1 | 0.7 |
|  | GAG | 391739 | 56.4 | 1.6 | 574785 | 47.1 | 1.4 | 500645 | 54.6 | 1.6 | 513307 | 46.0 | 1.4 |
| Cys | UGU | 50729 | 7.3 | 0.7 | 137799 | 11.3 | 1.0 | 73317 | 8.0 | 0.7 | 105967 | 9.5 | 0.8 |
|  | UGC | 93996 | 13.5 | 1.3 | 146260 | 12.0 | 1.0 | 135606 | 14.8 | 1.3 | 146246 | 13.1 | 1.2 |
| Trp | UGG | 76036 | 11.0 | 1.0 | 139354 | 11.4 | 1.0 | 114014 | 12.4 | 1.0 | 130405 | 11.7 | 1.0 |
| Arg | CGU | 32405 | 4.7 | 0.5 | 70615 | 5.8 | 0.6 | 38150 | 4.2 | 0.4 | 56116 | 5.0 | 0.5 |
|  | CGC | 96272 | 13.9 | 1.4 | 128959 | 10.6 | 1.1 | 148177 | 16.2 | 1.6 | 116152 | 10.4 | 1.1 |
|  | CGA | 34036 | 4.9 | 0.5 | 70368 | 5.8 | 0.6 | 37173 | 4.1 | 0.4 | 65047 | 5.8 | 0.6 |
|  | CGG | 81476 | 11.7 | 1.2 | 95604 | 7.8 | 0.8 | 117121 | 12.8 | 1.3 | 98650 | 8.8 | 0.9 |
|  | AGA | 65960 | 9.5 | 1.0 | 164300 | 13.5 | 1.5 | 72890 | 7.9 | 0.8 | 143725 | 12.9 | 1.4 |
|  | AGG | 107949 | 15.6 | 1.6 | 152020 | 12.4 | 1.3 | 135473 | 14.8 | 1.5 | 153012 | 13.7 | 1.5 |
| Gly | GGU | 61193 | 8.8 | 0.5 | 152588 | 12.5 | 0.8 | 73448 | 8.0 | 0.5 | 126438 | 11.3 | 0.7 |
|  | GGC | 170371 | 24.5 | 1.5 | 218461 | 17.9 | 1.2 | 258404 | 28.2 | 1.7 | 225428 | 20.2 | 1.3 |
|  | GGA | 96809 | 13.9 | 0.8 | 235965 | 19.3 | 1.3 | 118104 | 12.9 | 0.8 | 213969 | 19.2 | 1.2 |
|  | GGG | 137715 | 19.8 | 1.2 | 148368 | 12.1 | 0.8 | 173511 | 18.9 | 1.1 | 139917 | 12.5 | 0.8 |
| TER | UAA | 2666 | 0.4 | 0.8 | 6409 | 0.5 | 1.0 | 1640 | 0.2 | 0.6 | 4398 | 0.4 | 0.8 |
|  | UAG | 2458 | 0.4 | 0.7 | 3883 | 0.3 | 0.6 | 2047 | 0.2 | 0.8 | 3227 | 0.3 | 0.6 |
|  | UGA | 5564 | 0.8 | 1.6 | 8990 | 0.7 | 1.4 | 4127 | 0.4 | 1.6 | 8144 | 0.7 | 1.6 |

**Table S2. A complete list of positively selected genes in *Aldrovandia affinis*.**

| **Gene** | **Function** | **Adjusted *P* value** |
| --- | --- | --- |
| PIKFYVE | 1-phosphatidylinositol 3-phosphate 5-kinase | 3.20E-10 |
| DST | dystonin | 4.24E-08 |
| CENPF | centromere F-like | 1.64E-06 |
| EIF4B | eukaryotic translation initiation factor 4B | 1.82E-06 |
| CLASRP | CLK4-associating serine arginine rich protein | 1.96E-06 |
| UBAP2 | ubiquitin-associated 2 | 2.15E-06 |
| TAF1 | transcription initiation factor TFIID subunit 1 | 5.80E-06 |
| AGTPBP1 | cytosolic carboxypeptidase 1 | 1.03E-05 |
| CDK11B | cyclin-dependent kinase 11B | 2.11E-05 |
| BAG3 | BAG family molecular chaperone regulator 3 | 2.68E-05 |
| MTOR | serine/threonine-protein kinase mTOR | 2.70E-05 |
| RUBCN | run domain Beclin-1 interacting and cysteine-rich containing | 3.88E-05 |
| LENG8 | leukocyte receptor cluster member 8 homolog | 8.13E-05 |
| GTF3C1 | general transcription factor 3C polypeptide 1 | 1.29E-04 |
| COL18A2 | collagen alpha-2(I) chain | 1.82E-04 |
| PARP10 | poly ADP-ribose] polymerase 10 | 1.98E-04 |
| RCJMB04_719 | UPF0469 protein KIAA0907 homolog | 4.31E-04 |
| DNAJC4 | dnaJ homolog subfamily C member 4 | 1.20E-03 |
| SEC63 | translocation SEC63 homolog | 1.32E-03 |
| FBXL12 | F-box LRR-repeat 12 | 1.44E-03 |
| LRRC40 | leucine-rich repeat-containing 40 | 1.62E-03 |
| OBSL | obscurin-like | 2.26E-03 |
| DCK | deoxycytidine kinase | 2.55E-03 |
| STIM1 | stromal interaction molecule 1 | 2.69E-03 |
| PRDM9 | histone-lysine N-methyltransferase PRDM9 | 2.79E-03 |
| NIPB | nipped-B | 2.84E-03 |
| PRMT9 | putative arginine N-methyltransferase | 2.87E-03 |
| VPS16 | vacuolar sorting-associated 16 homolog | 2.88E-03 |
| CRTC1 | CREB-regulated transcription coactivator 1 | 3.49E-03 |
| ORC4 | origin recognition complex subunit 4 | 3.66E-03 |
| C10orf33 | Probable oxidoreductase C10orf33 | 3.75E-03 |
| RABGEF1 | Rab5 GDP/GTP exchange factor | 3.76E-03 |
| ANKRD12 | ankyrin repeat domain-containing 12 | 4.23E-03 |
| CCSER2 | serine-rich coiled-coil domain-containing protein 2 | 4.54E-03 |
| GOLGB1 | golgin subfamily B member 1 | 4.54E-03 |
| UPF2 | Regulator of nonsense transcripts 2 | 4.69E-03 |
|  | Neoverrucotoxin subunit alpha | 4.77E-03 |
| TTC37 | tetratricopeptide repeat 37 | 4.92E-03 |
| CWF19L2 | CWF19-like protein 2 | 4.98E-03 |
| P3H3 | prolyl 3-hydroxylase 3-like | 5.36E-03 |
| SMYD4 | SET and MYND domain-containing protein 4 | 5.75E-03 |
|  | hyccin | 6.54E-03 |
| DHX36 | ATP-dependent RNA helicase DHX36 | 7.51E-03 |
| RAB3GAP2 | rab3 GTPase-activating protein non-catalytic subunit | 7.67E-03 |
| HNRNPUL2 | heterogeneous nuclear ribonucleoprotein U-like protein 2 | 7.70E-03 |
| PAK6 | Serine/threonine-protein kinase 6 | 7.79E-03 |
| PCCB | propionyl-CoA carboxylase beta chain | 7.93E-03 |
| PPP5C | serine/threonine- phosphatase 5 | 7.94E-03 |
| ATXN2 | ataxin-2 | 8.29E-03 |
| GAB1 | GRB2-associated-binding 1 | 8.91E-03 |
| C21orf2 | protein C21orf2 | 1.04E-02 |
| CAPN5 | calpain-5 | 1.05E-02 |
| ALG5 | dolichyl-phosphate beta-glucosyltransferase | 1.06E-02 |
| TRMT1 | tRNA guanine26-N2-dimethyltransferase | 1.07E-02 |
| USP3 | ubiquitin carboxyl-terminal hydrolase 3 | 1.08E-02 |
| C12orf4 | C12orf4 homolog | 1.12E-02 |
| HINFP | histone H4 transcription factor | 1.22E-02 |
| PLEKHM2 | pleckstrin homology domain-containing family M member 2 | 1.31E-02 |
|  | M-phase phospho 8 | 1.46E-02 |
| ERCC4 | DNA repair endonuclease XPF | 1.55E-02 |
| RNF128 | E3 ubiquitin-protein ligase RNF128 precursor | 1.59E-02 |
| CLASP2 | CLIP-associating protein 2 | 1.71E-02 |
| CCDC94 | coiled-coil domain-containing protein 94 | 1.75E-02 |
| CEP350 | centrosome-associated protein 350 | 1.84E-02 |
| ULK3 | serine threonine- kinase ULK3 | 1.92E-02 |
| LPCAT3 | lysophospholipid acyltransferase 5-like | 1.99E-02 |
| AKAP13 | A-kinase anchor 13 | 2.20E-02 |
| ATG2A | autophagy-related protein 2 homolog A | 2.33E-02 |
| TECPR2 | tectonin beta-propeller repeat-containing 2 | 2.35E-02 |
| ZCCHC8 | zinc finger CCHC domain-containing 8 | 2.37E-02 |
| LIN37 | Protein Lin37 | 2.54E-02 |
| TGFB1I1 | transforming growth factor beta-1-induced transcript 1 protein | 2.55E-02 |
| ODF2L | outer dense fiber 2-like | 2.56E-02 |
| TCEA1 | transcription elongation factor A 3 | 2.58E-02 |
| PRELID1 | PRELI domain-containing protein 1 | 2.90E-02 |
|  | optineurin-like | 3.00E-02 |
| CACFD1 | calcium channel flower homolog | 3.04E-02 |
| TMEM181 | transmembrane protein 181 | 3.04E-02 |
| RF1C | replication factor C subunit 1-like | 3.09E-02 |
| NOP14 | nucleolar protein 14 | 3.18E-02 |
| PTCD3 | pentatricopeptide repeat domain-containing protein 3 | 3.45E-02 |
| COL18A1 | collagen alpha-1 (XVIII) chain | 3.48E-02 |
| PRCC | proline-rich protein PRCC | 3.49E-02 |
| HDX | highly divergent homeobox | 3.49E-02 |
| FRMD6 | FERM domain-containing protein 6 | 3.55E-02 |
| WDR33 | pre-mRNA 3 end processing | 3.56E-02 |
|  | DNA-directed RNA polymerase | 3.61E-02 |
| CDC7 | cell division cycle 7-related protein kinase | 3.84E-02 |
| EEA1 | early endosome antigen 1 | 4.11E-02 |
| FAM120B | constitutive coactivator of peroxisome proliferator-activated receptor gamma | 4.13E-02 |
| B4GALT4 | beta-1,4-galactosyltransferase 4 | 4.15E-02 |
| NUP153 | nuclear pore complex Nup153 | 4.30E-02 |
| RMDN1 | regulator of microtubule dynamics 1 | 4.32E-02 |
| FRYL | furry homolog-like | 4.32E-02 |
| WDR5 | WD repeat-containing 5 | 4.44E-02 |
| WBSCR27 | Williams-Beuren syndrome chromosomal region 27 | 4.45E-02 |
| STRN3 | striatin-3 | 4.85E-02 |
| DDR1 | epithelial discoidin domain-containing receptor 1 | 4.87E-02 |
| SFSWAP | splicing suppressor of white-apricot homolog | 4.88E-02 |
| GAPVD1 | GTPase-activating and VPS9 domain-containing 1 | 5.24E-02 |
| IGHMBP2 | DNA-binding SMUBP-2, DNA repair, | 5.25E-02 |
| CHTOP | chromatin target of PRMT1 | 5.29E-02 |
| ZRSR2 | U2 small nuclear ribonucleoprotein auxiliary factor 35 kDa subunit-related protein 2 | 5.72E-02 |
| RGL | ral guanine nucleotide dissociation stimulator-like | 5.75E-02 |
| LRCH3 | leucine-rich repeat and calponin homology domain-containing 3 | 5.76E-02 |
| DPF3 | zinc finger protein DPF3 | 5.91E-02 |
| RNF41 | E3 ubiquitin-protein ligase NRDP1 | 6.01E-02 |
| TMEM59 | transmembrane protein 59 | 6.02E-02 |
| MCT10 | monocarboxylate transporter 10 | 6.03E-02 |
| TDRD3 | tudor domain-containing protein 3 | 6.05E-02 |
| RNMT | mRNA cap guanine-N7 methyltransferase | 6.12E-02 |
|  | carboxylate synthase | 6.41E-02 |
| NUP98 | nuclear pore complex protein Nup98-Nup96 | 6.56E-02 |
| CEBPB | CCAAT/enhancer-binding protein beta | 6.61E-02 |
| UCHL3 | ubiquitin carboxyl-terminal hydrolase isozyme L3 | 7.28E-02 |
| IGF2R | cation-independent mannose-6-phosphate receptor | 7.29E-02 |
| GLTSCR2 | glioma tumor suppressor candidate region gene 2 protein | 7.30E-02 |
| PTPRQ | phosphatidylinositol phosphatase | 7.33E-02 |
| CDK5RAP2 | CDK5 regulatory subunit-associated 2 | 7.35E-02 |
| USP10 | ubiquitin carboxyl-terminal hydrolase 10 | 7.37E-02 |
| TER | Trans-2-enoyl-CoA reductase, mitochondrial precursor | 7.39E-02 |
| RPL28 | 39S ribosomal protein L28 | 7.39E-02 |
| RABEP1 | rab GTPase-binding effector 1 | 7.42E-02 |
| HPS4 | Hermansky-Pudlak syndrome 4 | 7.46E-02 |
| UHRF2 | E3 ubiquitin-protein ligase UHRF2 | 7.54E-02 |
| HIF1A | hypoxia-inducible factor-4 alpha | 7.61E-02 |
| COL5A2 | collagen alpha-2 (V) chain | 7.97E-02 |
| CKAP5 | cytoskeleton-associated 5 | 8.19E-02 |
| GCP3 | gamma-tubulin complex component 3 | 8.42E-02 |
| TK2 | Thymidine kinase 2, mitochondrial | 8.68E-02 |
| STON1 | stonin-1 | 8.72E-02 |
| TPA | tissue-type plasminogen activator | 8.92E-02 |
| RRP12 | RRP12 | 9.00E-02 |
| METTL16 | methyltransferase-like protein 16 | 9.28E-02 |
| GAPDH | glyceraldehyde-3-phosphate dehydrogenase | 9.61E-02 |
| PTX3 | pentraxin-related protein PTX3 | 9.64E-02 |
| VPS13A | vacuolar sorting-associated 13A | 9.65E-02 |
| GEMIN5 | gem-associated 5 | 9.69E-02 |

**Figure S1. Statistics of assembly contigs length.**
